# Lithium administered to pregnant, lactating and neonatal rats: entry into developing brain

**DOI:** 10.1186/s12987-021-00285-w

**Published:** 2021-12-07

**Authors:** Shene Yi-Shiuan Chiou, Kai Kysenius, Yifan Huang, Mark David Habgood, Liam M. Koehn, Fiona Qiu, Peter J. Crouch, Swati Varshney, Katherine Ganio, Katarzyna Magdalena Dziegielewska, Norman Ruthven Saunders

**Affiliations:** 1 Department of Biochemistry & Pharmacology, University of Melbourne, Parkville, VIC 3010 Australia; 2 Department of Neuroscience, Monash University, 99 Commercial Road, Melbourne, VIC 3004 Australia; 3 Bio21 Molecular Science and Biotechnology Institute, University of Melbourne, Parkville, VIC 3010 Australia; 4 Department of Microbiology and Immunology, The Peter Doherty Institute for Infection and Immunity, University of Melbourne, Melbourne, VIC 3000 Australia

**Keywords:** Pregnancy, Bipolar-disorder prophylaxis, Placental transfer, Postnatal, Brain barriers, Cerebrospinal fluid

## Abstract

**Background:**

Little is known about the extent of drug entry into developing brain, when administered to pregnant and lactating women. Lithium is commonly prescribed for bipolar disorder. Here we studied transfer of lithium given to dams, into blood, brain and cerebrospinal fluid (CSF) in embryonic and postnatal animals as well as adults.

**Methods:**

Lithium chloride in a clinically relevant dose (3.2 mg/kg body weight) was injected intraperitoneally into pregnant (E15–18) and lactating dams (birth-P16/17) or directly into postnatal pups (P0–P16/17). Acute treatment involved a single injection; long-term treatment involved twice daily injections for the duration of the experiment. Following terminal anaesthesia blood plasma, CSF and brains were collected. Lithium levels and brain distribution were measured using Laser Ablation Inductively Coupled Plasma-Mass Spectrometry and total lithium levels were confirmed by Inductively Coupled Plasma-Mass Spectrometry.

**Results:**

Lithium was detected in blood, CSF and brain of all fetal and postnatal pups following lithium treatment of dams. Its concentration in pups’ blood was consistently below that in maternal blood (30–35%) indicating significant protection by the placenta and breast tissue. However, much of the lithium that reached the fetus entered its brain. Levels of lithium in plasma fluctuated in different treatment groups but its concentration in CSF was stable at all ages, in agreement with known stable levels of endogenous ions in CSF. There was no significant increase of lithium transfer into CSF following application of Na^+^/K^+^ ATPase inhibitor (digoxin) in vivo, indicating that lithium transfer across choroid plexus epithelium is not likely to be via the Na^+^/K^+^ ATPase mechanism, at least early in development. Comparison with passive permeability markers suggested that in acute experiments lithium permeability was less than expected for diffusion but similar in long-term experiments at P2.

**Conclusions:**

Information obtained on the distribution of lithium in developing brain provides a basis for studying possible deleterious effects on brain development and behaviour in offspring of mothers undergoing lithium therapy.

**Supplementary Information:**

The online version contains supplementary material available at 10.1186/s12987-021-00285-w.

## Background

Lithium salts have been used since the eighteenth century to treat a wide variety of conditions and ailments [1]. It was introduced by Cade [2] for the treatment of bipolar disorder, although he did not use it for many of his patients because of concerns about toxicity [1]. It is now recognised that lithium is the “gold standard” treatment for bipolar [3, 4] and depressive disorders [5] and may be used in conjunction with a number of other drugs [6]. For reasons that seem unclear, lithium is prescribed for oral use in the form of lithium carbonate capsules with strict instructions that they should not be bitten [6] presumably because it is highly alkaline and would burn the mouth. Most published studies on lithium in rats have used the more neutral lithium chloride (LiCl, e.g. [7–9]) which we have also used in our studies. There does not appear to be a difference in terms of ionisation and pharmacokinetics between lithium chloride and lithium carbonate [10].

### Interactions between lithium and naturally occurring ions in permeability mechanisms

There is the long-established observation that lithium can substitute for sodium in cellular permeability mechanisms involving specific channels and ion exchange systems [11]. Lithium ions interact with sodium and to a lesser extent potassium, magnesium and calcium ions in channels and transport systems in a variety of tissues (e.g. [12–15]) but generalisations are difficult to make due to tissue differences [16]. There have been a few studies of lithium transfer across the mammalian choroid plexus involving experimental evidence for the exchange of lithium between blood and CSF, but disagreement exists about the mechanisms involved [17–20]. Recent studies investigating localisation and function of ion channels and transporters in choroid plexus epithelial cells, in both adult and during development [20–23] may eventually provide a better understanding.

### Lithium during pregnancy and lactation

Medications taken by pregnant and lactating women carry a risk to the fetus and neonate by potentially having untoward effects if transferred across the placenta into the fetal circulation or via breast tissue into the milk. Accordingly, many women, either pregnant or planning pregnancy, are advised to limit taking any medications to mitigate potential risks, some of which may still not have been identified [24, 25] as there is a dearth of evidence-based guidelines for clinical practitioners and patients regarding the safety of maternally administered drugs [26]. For women with bipolar and other psychiatric disorders that require long-term treatment, avoiding medications could result in more harm than good for both the mother and for the baby. Studies have shown that relapse of bipolar episodes are common when lithium treatment is discontinued [27, 28].

### Effects of lithium on fetal and neonatal development

There is only limited information on possible effects of lithium administered during pregnancy and breast feeding. Much of the available information is summarised in [29]. As with other drugs the focus has tended to be on whether lithium can induce teratogenic malformations in the fetus. The evidence is mixed because it is based mainly on retrospective uncontrolled studies of small numbers of patients. One prospective comparative study found no differences in rates of major congenital malformations between treated and control groups [30].

### Entry of lithium into the adult and developing brain

There have been several studies of lithium entry into the adult brain, mainly in rats [7–9, 31] but also a few in human patients [32, 33]. Two studies used imaging methods to study the distribution of lithium administered to adult rats [34, 35]. These and the quantitative studies of lithium measured in different brain regions all show that there is considerable regional variation in its cerebral distribution. These data will be considered in the Discussion in relation to the results of the present study.

In contrast to the adult, studies of lithium entry into the developing brain are lacking. For maternally administered lithium to enter the fetal brain it needs to: (i) cross the placental interface between maternal and fetal circulations; (ii) from the fetal circulation, lithium has to cross fetal brain barriers: the blood–brain barrier proper (between the blood and brain parenchyma across the cerebral blood vessels) and the blood-cerebrospinal fluid (CSF) barrier, which are the choroid plexuses [36]. In the case of breast feeding, for maternally ingested lithium to reach the postnatal brain it would need to enter the milk, cross the gut of the neonate and then cross its brain barriers to enter and distribute in its brain. In the present study an attempt was made to distinguish between various exchange interfaces (placenta, choroid plexus, brain, ingested breast milk) in order to gain a better understanding of how maternally administered lithium enters and distributes in the developing brain of the offspring. Entry of lithium by passive diffusion or some active transport mechanisms have been compared. Digoxin was used to inhibit sodium/ potassium ATPase in P4 pups, because of known interactions between lithium and sodium (see above). Entry of lithium into CSF at the ages studied was compared with previously published permeability data on markers of passive permeability by plotting CSF/plasma ratios (%) against molecular radius.

## Materials and methods

### Chemicals

Anaesthetics: Isoflurane (Pharmachem Queensland); Urethane > 99% (Sigma-Aldrich, Australia, CAS number 51–79-6).

Heparin: Heparin Sodium (Porcine Mucous) Injection BP (HameIn pharmaceuticals GmbH, Germany).

Lithium Chloride Anhydrous LR (Chem-Supply, CAS number: 7447-41-8).

Optimal Cutting Temperature media: TissueTek OCT from Sakura Finetek.

Isotonic sodium chloride: 0.9 g/100 ml.

Sodium Chloride (Baxter Healthcare, New South Wales).

Digoxin 98.9% purity (Sigma-Aldrich, CAS number: 20830-75-5).

### Animals and surgical procedures

Sprague Dawley rats were sourced from The Biomedical Sciences Animal Facility at The University of Melbourne. All rats were subjected to a 12 h light/ dark cycle with ad libitum access to food and water. All experimental procedures were conducted in accordance with National Health and Medical Research Committee (NHMRC) guidelines, the ARRIVE guidelines and approved by The University of Melbourne Ethics Committee, Ethics ID 1914793 (Entry of Anti-Epileptic and Psychotic Drugs into the Developing Brain).

As present experiments required administration of lithium by intraperitoneal (i.p.) injection, the more neutral lithium chloride salt rather than the strongly alkaline clinical preparation of lithium carbonate was used (see “Background” section).

Animals were randomly allocated to two experimental protocols:

(i) acute experiments, where animals received a single injection of lithium chloride (LiCl),
(ii) long-term exposure experiments where animals received multiple injections of LiCl over several days.

**Fig. 1:**
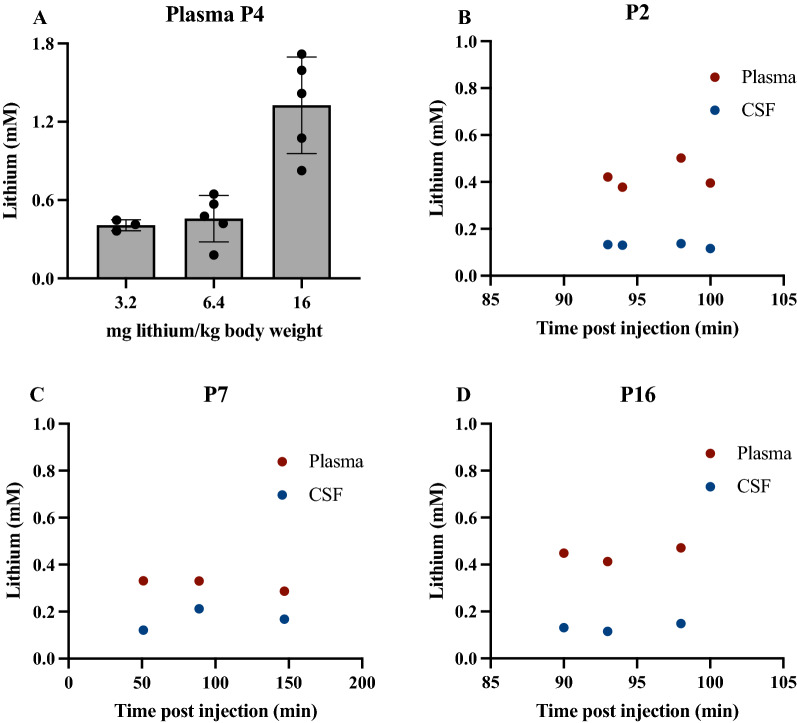
Determination of Injection Protocol. **A** Concentration of lithium in plasma of a litter of P4 rats administered lithium at concentrations of 3.2, 6.4 or 16 mg/kg body weight via i.p. injection, samples were collected 90 min after injection. Each dot represents an individual animal. Means ± standard deviation (SD). **B**–**D** Lithium 3.2 mg/kg body weight was injected i.p. into postnatal animals at the ages indicated; plasma and CSF were collected from pups at the post-injection times indicated. Each dot in **B**–**D** represents samples from individual animals; paired plasma and CSF for each pup are aligned vertically on graphs. Concentrations of lithium in un-injected control rats (P4) were below the limit of quantitation (LoQ).

Therapeutic concentrations of lithium in the blood of patients on lithium therapy are in the range of 0.4–1.2 mmol/l [6], expressed in the present study as mM. Doses and injection volumes were standardised to the animal’s body weight (3.2 mg/kg lithium, see Fig. 1A) to achieve the lowest therapeutic concentration of lithium in plasma and injection volumes were limited to < 1% of body weight to avoid any significant increase in the circulating blood volume. LiCl was dissolved in 0.9% sterile sodium chloride prior to intraperitoneal (i.p.) injection (Table 1).

**Table 1:** Ages and numbers of rats used

Age	P0	P2	P4	P7	P12	P16/17
A. Untreated fetuses/pups						
n	4 (2)	3 (2)	4 (4)	4 (2)	3 (3)	3 (2)
CR (mm) ± SD Weight (g) ± SD	5 ± 0.4	8 ± 2	9 ± 1	16 ± 2	29 ± 5	45 ± 2

Age	E18	P0	P2	P4	P7	P12	P16/17
B. Acute fetuses/pups							
n	22 (2)	3 (1)	8 (2)	43 (7)	9 (3)	6 (2)	8 (3)
CR (mm) ± SD Weight (g) ± SD	23 ± 1	5 ± 0.4	8 ± 2	9 ± 3	17 ± 3	25 ± 4	48 ± 4

Age	E18	P2	P4	P7	P12	P16/17
C. Long-term fetuses/pups						
n	23 (2)	2 (1)	6 (2)	8 (2)	1	7(2)
CR (mm) ± SD Weight (g) ± SD	22 ± 2	6, 8	11 ± 2	20 ± 1	40	50 ± 2

Treatment group	Untreated	Acute E18	Long term E18	Acute Breast-feeding	Long-term breast feeding
D. Dams					
n	1	2	2	2	2
CR (mm) ± SD Weight (g) ± SD	312	431, 316	350, 336	354, 336	352, 310

Ages, weights and numbers of animals used of A. untreated, B. acute, C. long-term treated fetuses/pups and D. Dams. E = embryonic, P = postnatal, n, number of animals at each age, SD = standard deviation. For sex and weight of individual animals see Additional file 1: Tables S1, S2. Numbers in brackets indicate the number of litters used for each age group. Note that for E18, the pup’s size is given in mm of Crown-Rump (CR) lengths as this is a more accurate parameter than weight for fetal staging.

Untreated animals were litter-mates of treated animals (acute experiments) or were obtained from other studies, including untreated control adult females [37, 38]. All individual lithium measurements are shown in Additional file 1: Tables S3–S5.

### Injection protocols

#### Fetal animals

Fetal animals were exposed to lithium via placental transfer only. This was achieved by i.p. injections into time-mated pregnant dams. For acute exposure experiments, E18 pregnant rats were administered a single i.p. injection of 3.2 mg/kg lithium. For long-term exposure experiments, twice daily 3.2 mg/kg lithium i.p. injections (early morning and late afternoon) were administered to dams from E15–E18.

#### Postnatal animals

For acute experiments, individual pups and dams were injected i.p. with 3.2 mg/kg lithium and left for 90–120 min (duration where plasma and CSF levels are stable, see Fig. 1B–D). For long-term experiments, pups were exposed to lithium only via breast milk from dams injected twice daily (early morning and late afternoon) with a standard dose of lithium (3.2 mg/kg body weight) and collected after 2, 4, 7, 12 or 16/17 days of exposure to breast milk. Dams were collected together with P16/17 pups (17 or 18 days of treatment respectively). Pups in the long-term experiments were separated from the dam 2–3 h after dam’s last injection.

Untreated control animals were age-matched to all experimental animals.

### Treatment protocols and surgery

#### Fetal animals

Pregnant rats were deeply anaesthetised (urethane 2.5 g/kg i.p.), a tracheal cannula inserted to maintain the airway and left femoral artery catheterised for collection of maternal blood samples. Animals were placed on heated plate (33ºC) and a uterine horn exteriorised via a mid-line abdominal incision. Pups, starting at the ovary end of the uterine horn, were removed from their amniotic sac and samples collected as described below. Any pups with visible signs of haemorrhages were not collected, but their position in the uterine horn was recorded.

#### Postnatal animals

Pups were terminally anaesthetised with an overdose of inhaled anaesthetic (> 5% isoflurane), before samples were collected (see below).

### Inhibition of sodium/ potassium ATPase in P4 pups

Pups at P4 were used for the experiment investigating if partial blocking of Na^+^/K^+^ ATPase could influence lithium transfer into the CSF. Seven pups from two separate litters (3 and 4 respectively) were injected with digoxin (300 mg/kg body weight in 75 µl, dissolved in ethanol followed by isotonic sodium chloride solution) and 7 matched littermates were injected with equal volume of isotonic sodium chloride solution, all injections were given i.p. Pups were carefully monitored for 30 min, before they were given the same lithium injection protocol as other postnatal animals (see injection protocol). Samples were all collected within 90–105 min after lithium injection and all samples were coded for blinded determination of lithium analysis (see below).

### Collection and processing of blood and CSF samples

Blood samples were taken directly from the right ventricle of the heart into heparinised glass capillaries. For the acute experiments in pregnant rats, a small sample of maternal arterial blood (0.2 ml) was collected from the femoral arterial catheter at the same time as each pup was removed and the catheter then flushed with an equal volume of isotonic sodium chloride solution to maintain circulating volume (~ 2 ml of blood was collected from each dam in total).

Blood samples were centrifuged for 5 min (2000×*g*) and plasma separated. Samples of CSF were collected from the cisterna magna using a glass micropipette, with care taken to avoid rupturing blood vessels. Any sample that was visibly contaminated with blood was discarded (contamination as small as 0.2% can be visibly detected [39]. Plasma and CSF samples were quantified using two different methods: inductively coupled plasma-mass spectrometry (ICP-MS) and laser ablation-inductively coupled plasma-mass spectrometry (LA-ICP-MS).

### ICP-MS

Frozen aliquots of undiluted plasma and CSF were thawed at room temperature. Plasma (100 μl in adults and 10–20 μl for E18 and P4 animals) and 10 µl of CSF were aliquoted into separate 1.7 ml microcentrifuge tubes. Plasma and CSF were diluted in 3.5% (v/v) nitric acid (HNO_3_, 70% Analytical grade from Ajax Finechem) in Milli-Q H_2_O (18.2 MΩ; Merk Millipore, Australia) (1:10 and 1:100 respectively to a final volume of 1 ml) and digested by heating to 90 °C for 10 min. Samples were briefly vortexed and centrifuged at 20,000×*g* for 20 min and supernatant was immediately transferred to a new 1.7 ml microcentrifuge tubes. Blanks were prepared in an identical manner and 1 ml of 3.5% HNO_3_ blanks were also aliquoted for analysis.

### LA-ICP-MS

Plasma samples (0.5 μl) diluted in 0.9% NaCl, 1:10 v/v and undiluted CSF were spotted in onto a glass slide in triplicate (Superfrost glass slides, Thermo Scientific) and air dried for 24 h before analysis.

### Collection and processing of brain samples

Whole brains, including olfactory lobes, were dissected out of the skull and bisected along the sagittal plane into two equal halves. Each half was randomly assigned to either extraction and quantification of lithium (using ICP-MS or LA-ICP-MS) or sectioning (for LA-ICP-MS).

#### Lithium extraction for ICP-MS

Approximately 50mg of tissue was transferred to a 1.7 ml microcentrifuge tube and lyophilised. Both the wet weight and dry weight of the tissue were measured to report elemental concentration in terms of g of wet and dry tissue weight. Lyophilised tissue was digested in 50 µl of 70% (v/v) HNO_3_ (70% Analytical grade from Ajax Finechem) at 90 °C for 20min. Samples were allowed to cool prior to addition of 50 µl of 30% (v/v) hydrogen peroxide (H_2_O_2_) (30% Hydrogen Peroxide, Analytical grade from Merck) and heated to 70 °C for 15min. Samples were allowed to cool then diluted to a final volume of 1ml with 1% (v/v) HNO_3_ in Milli-Q H_2_O. Samples were briefly vortexed and centrifuged at 20,000×*g* for 25min and the soluble material was immediately transferred to a new 1.7 ml microcentrifuge tube. Preparation blanks were prepared in an identical manner. Water blanks were also taken during the measurement.

### Inductively coupled plasma-mass spectrometry (ICP-MS)

An Agilent 8900 triple quadrupole ICP-MS (Agilent Technologies) was tuned and optimised using a tuning solution containing 1 μg/l of cerium (Ce), cobalt (Co), lithium (Li), thallium (Tl) and Y in 2% (v/v) HNO_3_ (Agilent Technologies, Australia). The ion intensity at m/z 7 (lithium) was monitored in a no collision gas analysis mode. The instrument was calibrated using a 10-point calibration curve for lithium using commercially available multi-element standards at 0, 1, 5, 10,25, 50, 100, 250, 500 and 1000 parts per billion (ppb) in 1% HNO_3_ (Multi-Element Calibration Standard 2A, Agilent Technologies, USA). Yittrium (89Y) (Agilent Technologies) was used as an internal reference elemental standard at a concentration of 0.1 μg/ml and used to normalise recovery across all samples. All samples, calibration standards and internal standards were introduced to the nebuliser using a peristaltic pump and T-piece for sample mixing at the flow rate of 0.4 ml/min. The ICP-MS operating parameters were established according to the manufacturer’s guidelines and other parameters were optimised for lithium in a batch specific mode prior to each experiment are as follows:

**Table:** 

ICP-MS operating parameters	
Scan type	Single Quad
RF Power	1550 W
RF Matching	1.2 V
Nebulizer Gas	1.09 l/min
Extract 1	− 12 V
Extract 2	− 215 V
Omega Bias	− 95 V
Omega Lens	5.2 V
Deflect	10.8 V
Collision Gas	No Gas
Oct P bias	− 8 V

Limits of detection (LOD: 0.013 μg/l) and quantification (LOQ: 0.083 μg/l) calculated as 1 × SD of 10 replicate blanks were obtained. Seronorm™ Trade Elements Serum L-1 and L-2 (Sero, Norway) were used to externally assess analytical performance and sample recovery was within ± 20% RSD of manufacturer’s expected values.

#### Lithium extraction for LA-ICP-MS

Half of the brains designed for the extraction were frozen on dry ice and stored at − 80 $^\circ \mathrm{C}$ until use. Brain samples were defrosted for a minimum of 30 min at room temperature, homogenised in isotonic sodium chloride solution (1:2 w/v) using a glass pestle, mixed vigorously and incubated at 37 $^\circ \mathrm{C}$ for 20 min. Samples were mixed briefly, centrifuged for 10 min (2000×*g*) and supernatant collected (total extracted volumes were recorded). The supernatants were diluted with isotonic sodium chloride (1:5 and 1:10 v/v) and replicates spotted onto glass slides, air dried and analysed using LA-ICP-MS (see below).

### Laser ablation-inductively coupled-mass spectrometry (LA-ICP-MS)

*Sectioning of brain for LA-ICP-MS*: the frozen half brain samples (see above) were immediately placed in a mould containing Optimal Cutting Temperature media (OCT), frozen on dry ice and stored at − 80 $^\circ \mathrm{C}$ until sectioning. Frozen brain blocks were temperature-equilibrated for at least an hour before sectioning (30 µm serial sagittal sections) in a cryostat. Sections were mounted on glass slides and air dried overnight before LA-ICP-MS analysis.

Samples (0.5 l μl “droplets” or 30 μm brain sections) were air dried on standard microscope slides for 24 h before being placed into a 10 × 10 cm ablation cell where the laser was tuned to the topography and dimensions of the sample slides together with matrix-matched elemental standards for quantitative analysis [40]. Brain sections were ablated with a 213 nm laser (NWR213 ablation system, Kennelec Scientific) by a series of rasters using a 60 × 60 μm square spot size and a scanning speed of 240 μm/s for low resolution scans, and 30 × 30 μm square spot size and a scanning speed of 120 μm/s for higher resolution scans. For droplet (CSF, plasma and brain homogenate) analysis 100μm^2^ spot size and speed of 200 μm/s was used. Ablated material was swept into Agilent 8800 QQQ-ICP-MS (Mulgrave, Victoria, Australia) by argon gas flow at 1.2 l/min and directed through the plasma torch for ionisation. Ionised material was analysed for lithium (^7^Li), carbon (^13^C), sodium (^23^Na), phosphorus (^31^P), potassium (^39^ K) and iron (^56^Fe) for a dwell time of 0.045 s per element. Carbon and phosphorus were recorded for tissue structure identification and iron for detection of residual blood in the tissue and CSF. Approximate analysis time for each brain section was 5−6 h and for each microscope slide of droplets (100–150 droplets on average) 20−24 h. Two dimensional elemental maps were constructed using the Iolite analysis software (School of Earth Sciences, University of Melbourne) operating under the Igor Pro suite [41].

As there are no polyatomic interferences for lithium [42], good limits of detection (LoD; 4.014 μg/l calculated as 3 × SD of 8 replicates blank) and quantitation (LoQ; 13.381 μg/l calculated as 10 × SD of 8 replicates blank) were obtained.

**Fig. 2:**
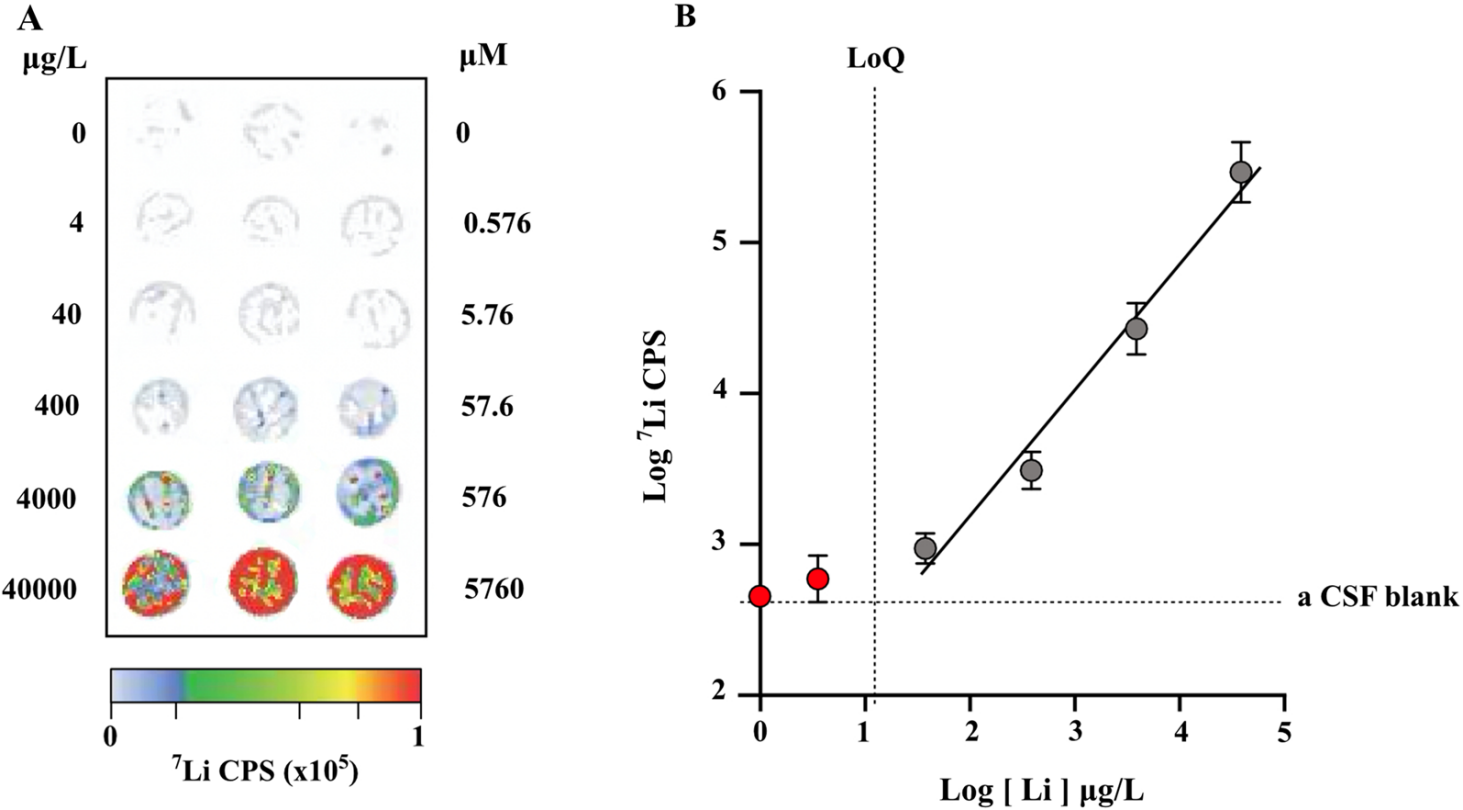
Laser Ablation Inductively Coupled Plasma-Mass Spectrometry. A. Representative elemental map of lithium standards in CSF matrix residues (µg/l and µM). B. Resulting standard curve for these samples. Lithium standard concentration curves were prepared independently for each experiment

Certified ICP-MS standards and LiCl-based standards were used for droplet lithium quantitation. Certified ICP-MS standards (Agilent) of 500 ppb (or µg/l) and LiCl of 240 mg/ml were serially diluted in a matrix-matched artificial CSF solution (148 mM NaCl, 3 mM KCl, 1.2 mM MgCl_2_, 10 mM glucose, 1 mg/ml bovine serum albumin) (Fig. 2) [43].

From the scans, regions of interest (each spot of plasma, CSF or brain homogenate sample) were selected and average counts per second for each residue were measured and calibrated to standard curve constructed from standards on the same slide. Information on the standards and quantitation used for brain sections and droplets can be found in [43, 44] respectively. All droplets were analysed in technical triplicates.

Note that there was reasonable concordance in the results from the two methods used for measuring lithium in the samples (Fig. 4). However, there was a general tendency for LA-ICP-MS to give higher values, a difference which was more obvious for some of the postnatal brain results (Fig. 6). It is likely that the explanation for these differences lies in the different extraction methods used.

### Experimental design

#### Randomisation

In acute experiments, littermates were randomly assigned to the untreated control and experimental groups. In long-term experiments, because all pups within the one litter were exposed to lithium via the mother, age-matched separate litters from untreated dams were used as controls.

#### Blinding of the study

Analysis of plasma, CSF and brain sections by LA-ICP-MS: samples were identified only by code which was decoded at the end of the experiment. Samples for ICP-MS were also number-coded before being processed and only decoded at the end of the process.

#### Statistical analysis

Statistical differences between levels of lithium in plasma, CSF and brain as well as CSF/plasma and brain/plasma ratios in acute and long-term treatment group at each age were determined using one-way ANOVA with Tukey’s posthoc test for multiple comparisons (Graphpad Prism 9).

## Results

Therapeutic concentrations of lithium in the blood of patients on lithium therapy are in the range of 0.4−1.2 mM [6]. In order to achieve similar concentrations in the animal model, an injection protocol was established. The first step was to find an appropriate dose, followed by establishing an experimental timeframe that would be consistent across all ages.

### Determining lithium concentrations in rat blood plasma

**Fig. 3:**
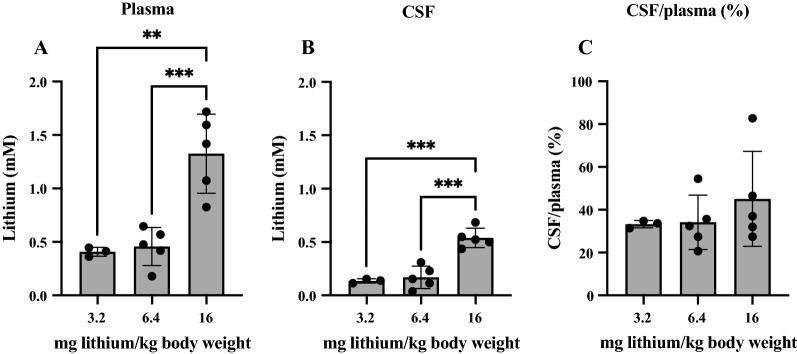
Concentrations of lithium in plasma and CSF. A. Plasma (same data as Fig. 1A). B. CSF and C. CSF/plasma ratios (%) of P4 rats administered lithium 3.2, 6.4 or 16 mg/kg body weight via i.p. injection. Note values are from LA-ICP-MS measurements, each dot in A and B represents an individual animal. CSF/plasma ratios (%) are calculated for each animal. Means ± SD. Differences in mean CSF/plasma ratios for different doses of lithium were not statistically significant, p = 0.05. Concentration of lithium in un-injected rats (P4): 4-13 µM. **p < 0.01, ***p < 0.001

Three doses of lithium (3.2, 6.4 and 16 mg/kg body weight) were injected i.p. into individual P4 pups from the same litter, pups left for 1.5 h, then terminally anaesthetized and blood and CSF samples collected as described in Methods. Lithium concentrations in each individual pup’s plasma, CSF and CSF/plasma ratios are illustrated in Fig. 3 (Additional file 2: Fig. S1).

In all animals the concentrations of lithium in CSF were lower than in plasma, but reflected the dose administered (Fig. 3B); CSF/plasma ratios were similar (around 25–45%) in all pups regardless of the injected dose (Fig. 3C). As can be seen in Fig. 3A the dose of 3.2 mg lithium/kg body weight was sufficient to achieve the lowest concentration (around 0.4 mM) that was within the clinical range of 0.4–1.2 mM [6]. This dose was used for the rest of the study.

### Establishing the experimental timeframe for postnatal animals

Levels of injected markers in an animal’s circulation are inherently variable therefore an established method for determining the entry of markers into CSF is to express the concentration in CSF as a ratio of that in plasma when at steady-state [45]. The steady-state CSF/plasma ratio is defined as the period when both CSF and plasma concentrations are relatively stable. In order to establish this period in rats at different developmental ages a litter-based model approach was used [39]. Standardised amounts of lithium (3.2 mg/kg body weight) were injected i.p. to a litter of P4 pups and samples of blood and CSF collected at different time points from 30 to 170 min (see Additional file 2: Fig. S1).

The period when the CSF/plasma ratio appeared stable was between 90 and 150 min (Fig. 1).

In order to establish if this time frame also applies to animals at different postnatal ages, a similar experiment was conducted in a limited number of pups at P2, P7 and P16. Results are illustrated in Fig. 1.

As shown in Fig. 1 the CSF/plasma ratios at the postnatal ages studied were relatively stable over the time up to 150 min after an i.p. injection of lithium. Therefore, a dose of 3.2 mg/kg body weight and an experimental timeframe of 90–120 min was used for all subsequent experiments.

For most experiments, samples from separate litters were measured using both methods (ICP-MS or LA-ICP-MS) and this is indicated by different coloured symbols in figures.

### Entry of lithium from blood plasma into the CSF in acute experiments

**Fig. 4:**
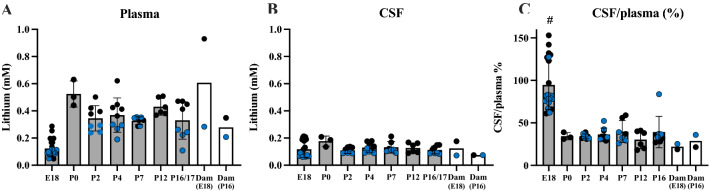
Concentration of lithium after acute exposure. **A** Plasma, **B** CSF and **C** CSF/plasma ratios (%) of animals following a single i.p. dose of lithium (3.2 mg/kg body weight). Means ± SD when n > 2. Blue points are samples measured using ICP-MS and black points are samples measured using LA-ICP-MS, each point is an individual animal. #Significantly different from all other groups. Ages in brackets refer to age when fetuses/pups were sampled

Rats at different developmental ages (dams at E18, pups at P0, 2, 4, 7, 12, 16/17 and a postnatal dam at P16) were injected i.p. with 3.2 mg/kg lithium and blood, CSF and brain samples collected within 90-120 min (Fig. 4). Each dot in in Fig. 4 represent samples from individual animals with CSF/plasma ratios (%) calculated for the same animal. Error bars are ± SD when n > 2. For untreated control values, see Additional file 1: Table S3. The E18 pups received lithium via placental transfer only.

As shown in Fig. 4A, plasma lithium concentrations in pups (P0-P16/17) following a single i.p. injection remained within the therapeutic window in plasma (~ 0.4 mM). In the E18 fetuses the plasma concentration was lower than in the postnatal animals. This reflects a degree of placental protection, but may also be partly because the dose of lithium was administered i.p. to the dam and would have distributed to all of the fetuses resulting in a larger distribution volume and therefore contribute to the lower concentration. CSF concentrations across ages were also stable at ~ 0.15–0.2 mM. CSF/plasma ratios were similar at all postnatal ages, including the dam (~ 35–40%, Fig. 4C) with the exception of E18 where ratios were much higher (~ 100%) because of the lower plasma value (Fig. 4A). Individual lithium sample values are shown in Additional file 1: Table S4.

### Entry of lithium from blood plasma into the CSF in long-term experiments

**Fig. 5:**
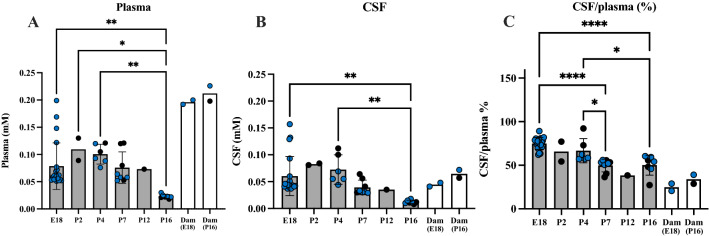
Concentration of lithium after long-term exposure. **A** Plasma, **B** CSF and **C** CSF/plasma ratios (%). Rats exposed to long-term lithium treatment via placental transfer (estimated at E18) or via breast milk (estimated in plasma and CSF at P2–P16) from treated dams. P0 is not included as in long-term treated pups the dam’s treatment started from the day of birth (P0). The lithium concentrations in plasma and CSF these long-term treated animals were less than in the acute experiments and declined with age (cf Figs. 4, 5). Each dot in A and B represents a sample from individual animals. CSF/plasma ratios (%) are calculated for the samples from the same animal. Blue points are samples measured using ICP-MS and black points are samples measured using LA-ICP-MS. Error bars are ± SD when n > 2. For untreated control values, see Additional file 1: Table S3. Note ages in brackets refer to age of fetuses/pups when dams were sampled at the same time. * p < 0.05, **p < 0.01, ****p < 0.0001

To compare results from pups exposed acutely to lithium to those exposed to lithium over a prolonged period of time, lithium was administered by i.p. injections to the dam over the course of pre- and post-natal rat development (E15–E18 and P0–P16 respectively, see Methods). This was in order to mimic clinical situations where pregnant and lactating mothers need to take daily lithium. Fetuses were exposed to lithium via placental transfer from the maternal circulation; postnatal animals were exposed via breast milk. Concentration of lithium was estimated using both, ICP-MS and LA-ICP-MS methods. The results are illustrated in Fig. 5.

The plasma and CSF concentrations of lithium in postnatal pups that received lithium via breast milk of treated dams were much lower than those acutely treated (see Fig. 4) at around 0.1 mM up to P4 after which it declined (acute vs long-term plasma and CSF: P < 0.01 and P < 0.05 respectively across all ages). In contrast to stable CSF lithium levels in acute experiments (see Fig. 4) in animals exposed to lithium over a prolonged time, concentration of lithium in the CSF appeared to decline with increasing age of the pups falling from 0.06 mM at E18 and 0.08 mM at P2 to 0.012 mM at P16 (Fig. 5B). This decline in CSF lithium concentration was reflected in similar age-dependent decline in CSF/plasma ratios, which ranged from 75% at E18 to 54% at P16 (p < 0.0001, Fig. 5C). The adult CSF/plasma ratio for lithium appeared to be lower than in the pups but this is most likely a reflection of higher concentrations in plasma (Fig. 5A) but similar concentrations in the CSF (Fig. 5B). The plasma levels were closer to the therapeutic range (about half) compared to the levels in the postnatal animals (about one quarter) perhaps indicating a degree of protection by the breast tissue. Statistical analysis was not performed for the results from dams due to their small numbers (but also see “Discussion” section). Individual lithium sample values are shown in Additional file 1: Table S4.

### Entry and distribution of lithium into the developing brain

Entry of lithium into the developing brain was also investigated using: (i) extraction of brain samples to obtain overall quantitative estimates of its concentration compared to plasma by ICP-MS and LA-ICP-MS, and (ii) LA-ICP-MS to map its distribution and obtain regional estimates of concentrations of lithium. Untreated control, age-matched brain samples were also included (Additional file 1: Table S3).

#### (i) Quantitative estimates of lithium in the brain

To quantify the amount of lithium in brain samples, lyophilised or homogenised brains were prepared as described in Methods. The results are presented in Table 2.

**Table 2:** Concentration of lithium in the brain

	E18	P0	P2	P4	P7	P12	P16	Dam (E18)	Dam (P16)
Acute experiment (mM)	0.05 ± 0.01 n = 16	0.08 ± 0.05 n = 3	0.40 ± 0.46 n = 6	0.05 ± 0.16 n = 6	0.13 ± 0.08 n = 7	N/A	0.17 ± 0.16 n = 6	0.12 n = 1	0.25 (0.45, 0.05)
Long term experiment (mM)	0.05 ± 0.03 n = 20	N/A	0.17 (0.15, 0.19)	0.09 ± 0.07 n = 6	0.04 ± 0.02 n = 8	0.12 n = 1	0.03 ± 0.02 n = 7	0.17 (0.19, 0.14)	0.31 (0.40, 0.23)

Average concentrations of lithium in the brain from acute and long-term treatment groups. P0 not included in long-term experiments (N/A) as the dam’s treatment started from day of birth. Individual values are in Additional file 1: Table S4. Untreated control values are in Additional file 1: Table S3. Note values from P0 and P12 experiments are from LA-ICP-MS brain homogenates only. All others are using both methods with a combined mean, n is number of individual pups. Values are means ± SD; where n = 2 both values are listed below the mean in brackets.

The concentration of lithium in brains from control untreated animals was also measured and was barely detectable in all postnatal pups (Additional file 1: Table S3) while in adults it was estimated as 0.0037 $\pm$ 0.01 mM (n = 8). Values for lithium in brains from acute experiments are from the same animals as illustrated for plasma and CSF in Fig. 4 and for long-term experiments the brain values are from the animals presented in Fig. 5.

In acutely treated animals, concentrations of lithium in the brain were consistently higher than in animals exposed to lithium via breast milk. In the fetuses, in contrast, both acute and long-term treated groups had similar and very low concentrations (~ 0.05 mM).

**Fig. 6:**
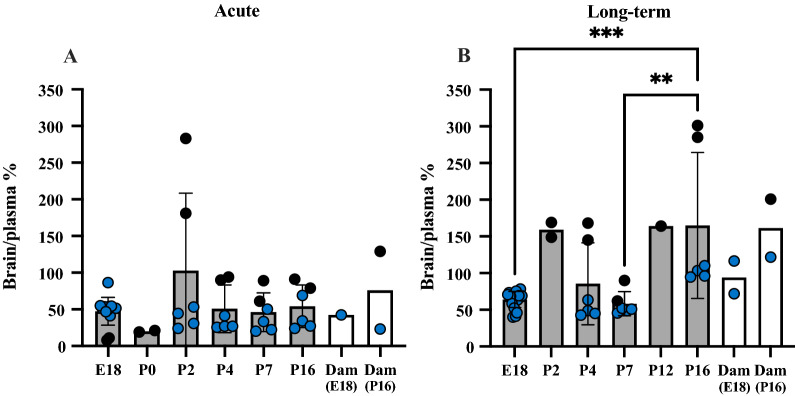
Brain/plasma lithium concentration ratios (%). **A** Acute and **B** long-term experiments. Brain/plasma ratios (%) estimated from values of the same animal as those shown in Figs. 4 and 5. P0 is not included in **B** as in long-term treated pups the dam’s treatment started from the day of birth. Note each point is an individual animal, blue points are samples measured using ICP-MS and black points are samples measured using LA-ICP-MS. Ages in brackets refer to age of fetus/pups when dams were also sampled. Means ± SD when n > 2, **p < 0.01, ***p < 0.001

To assess how entry of lithium into the brain was related to its concentration in plasma, brain to plasma concentration ratios (%) were calculated and results are shown in Fig. 6.

In the acutely treated animals, the brain/plasma ratios were relatively stable (~ 50%) from E18 fetuses to adults (dams). At each age brain ratios in postnatal pups from P2 onwards were higher than their corresponding CSF/plasma ratios (cf Figs. 4 and 6). The higher brain ratio reflects higher brain content of lithium, as the plasma sample values, used to calculate the ratios were the same for CSF and brain. In contrast, in the fetal pups at E18 CSF/plasma ratios were around 100% (Fig. 4C) while brain/plasma ratios were below 20% (Fig. 6A). Possible explanations for this difference are considered in “Discussion” section.

In long-term treated pups, brain/plasma ratios are illustrated in Fig. 6B. At P2, P4, P12, P16 and in the dams their brain/plasma ratios were close to 100–150% but clearly below 100% at E18 and P7. There were statistically significant differences between E18 and P16, p < 0.001 and P7 and P16, p < 0.01, Fig. 6B).

#### LA-ICP-MS analysis of brain sections

**Fig. 7:**
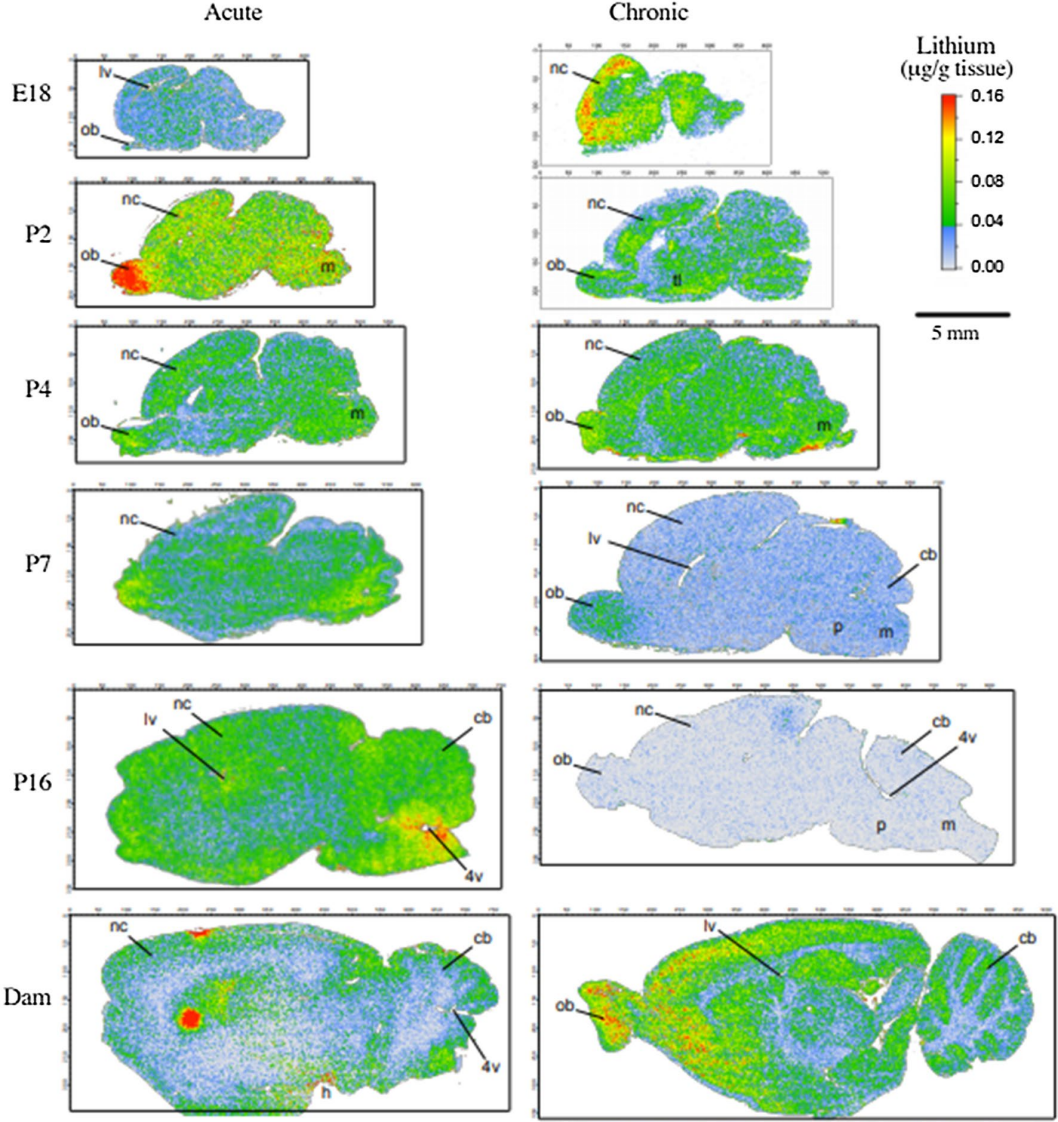
Distribution of Lithium in the brain following acute or long-term exposure to lithium at different ages. Representative sagittal sections of brains at different ages from animals exposed to lithium acutely (left hand panels) and long-term (right hand panels). Olfactory bulbs are to the left in all sections. The dimensions of the brain sections are approximately to scale but due to dehydration in preparation of tissue they can vary between ages. At most postnatal ages there is a concentration of lithium in the olfactory bulbs and more generalised lower-level distribution in the rest of the brain. There was a similar distribution in the postnatal brains of animals treated long-term, but with lower intensity. Control sections were always blank. More sections of treated and control brains are in Additional file 3: Fig S2. P0 is not included in the long-term treated group (right hand panels) as in long-term treated pups the dam’s treatment started from the day of birth. Labels: 4v (4^th^ ventricle), cb (cerebellum), h (hypothalamus), lv (lateral ventricle, m (medulla), nc (neocortex) ob (olfactory bulb), p (pons)

A summary of the distribution patterns of lithium in brain samples is shown in Fig. 7; other scans obtained, including control scans are in Additional file 3: Figure S2.

Lithium in both acute and long-term treated pups showed a regional pattern of localisation, with a distinct accumulation in the olfactory bulb, already at E18 but more prominent in postnatal ages particularly in the brains of acutely treated animals. In the rest of the brain there was a more generalised distribution. The concentration of lithium in the brains of pups that received lithium via breast milk following i.p. injection to the mother was much less at all ages than in acutely exposed pups which received an i.p. injection. In addition, the concentration of lithium in the brain decreased in long-term exposed pups as they got older and it was barely above background by P16. These differences between the acute and longer-term experiments are probably a consequence of the lower concentrations of lithium in plasma in breast-fed pups (Figs. 5, 6).

### Protective Function of the Placenta

**Fig. 8:**
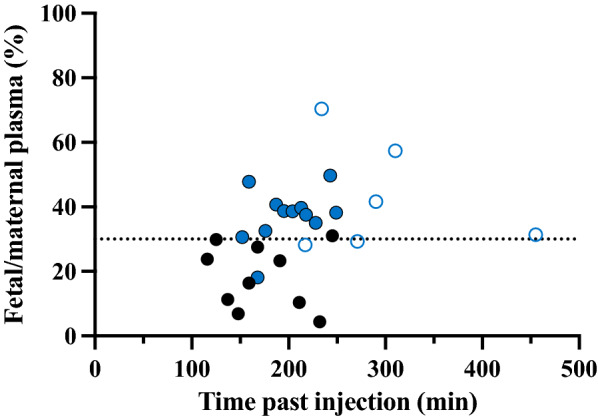
Fetal/maternal plasma lithium concentration ratios at E18. Closed symbols indicate acute treatment and open symbols indicate long-term treatment. Black symbols are samples measured using LA-ICP-MS and the blue symbols are ones measured using ICP-MS. Each point on the graphs represents a ratio calculated using fetal plasma levels in an individual fetus and the corresponding time-matched plasma of the dam.

To measure the degree of protection provided by the placenta, concentrations of lithium in the fetal plasma were compared to maternal plasma from dams that received either a single i.p. injection of 3.2 mg lithium/kg body weight at E18 (acute experiment) or twice daily i.p. injections of 3.2 mg lithium/kg body weight from E15 to E18 (long-term experiment). Both are illustrated in Fig. 8. Maternal blood samples were collected from the cannula inserted into the femoral artery (see “Methods” section) and were time-matched to each pup as it was removed from its amniotic sac.

**Fig. 9:**
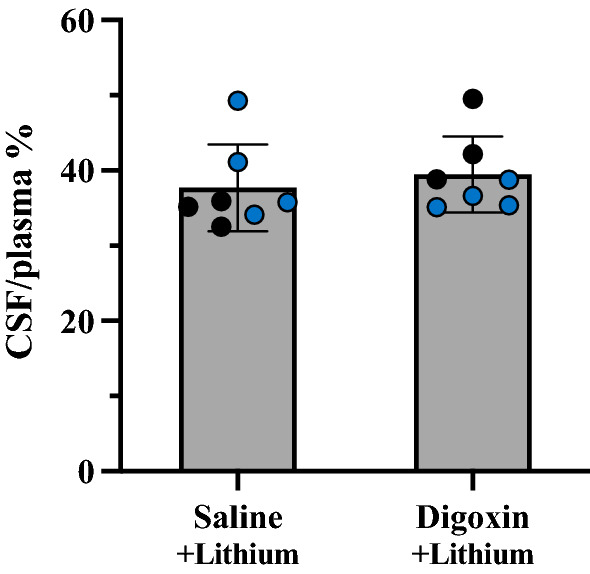
Effect of digoxin on entry of lithium into CSF of P4 rats. CSF/plasma ratios (%) of P4 littermates acutely exposed to lithium (3.2 mg/kg body weight) following treatment with a large dose of digoxin (300 mg/kg body weight) compared to isotonic sodium chloride vehicle. Each dot represents a sample from individual animals taken from 2 litters, black points measured using LA-ICP-MS and blue points measured using ICP-MS. Means ± SD.

As can be seen in Fig. 8, the ratio of fetal to maternal plasma lithium concentration in acute experiments ranged between different pups from about 10% to around 50% and was not related to the length of exposure. A similar ratio (37%) was obtained from the long-term treated dam where a final maternal sample was taken at 7 h indicating that placental transfer does not change much with time. This means that following administration to the dam more than 60% of maternal lithium did not reach the fetal circulation indicating that the placenta appears to be providing a substantial level of protection for the fetus. Nevertheless, 40% of the injected dose was reaching the fetus and much of this was reaching the developing brain (Table 2 and Figs. 7, 8, 9) and its CSF although to a lesser extent (Figs. 5, 6).

#### Lithium entry into the developing brain following inhibition of sodium/ potassium ATPase pump

To examine whether Na^+^/K^+^ ATPase pump is involved in lithium entry into the developing brain, digoxin, an inhibitor of the enzyme, was injected i.p. to P4 pups. Two litters were randomised and 3–4 pups from each litter received digoxin 30 min prior to an i.p. injection of lithium, while another 3–4 that served as control pups received an equal volume of isotonic sodium chloride for the same duration of time (Fig. 9, individual sample values are shown in Additional file 1: Table S5). There was no significant difference in CSF/plasma ratios (p > 0.05) between the two treatment groups. This lack of effect suggests that Na^+^/K^+^ ATPase may not play a functionally significant role in entry of lithium into the CSF early in brain development (see “Discussion” section).

## Discussion

This study aimed to measure the distribution and extent of lithium entry into the fetal (E18), neonatal (P0–17) and adult rat brain, following administration of clinically relevant doses of lithium either to pregnant and lactating mothers or to postnatal animals directly. This approach allows determination of the degree of protection provided by tissue barriers such as the placenta and breast tissue as well as the protection provided by individual blood brain barriers during rat development. In longer-term treatment experiments injections (i.p.) were made into the pregnant and lactating mothers to mimic the clinical situation of continuous maternal treatment and provide an estimate of placental and breast tissue protection. Acute experiments (single dose i.p.) were used to study in adult and developing animals the entry of lithium across the blood–brain and blood-CSF barriers separately.

### Transfer of maternally administered lithium across the placenta

The extent of protection provided by placenta against lithium entry into the fetus was assessed from lithium concentration ratios between fetal and maternal plasma. E18 is approximately equivalent to 22–24 weeks gestation in humans [46, 47] corresponding to the earliest age of viability [48, 49]. After acute maternal administration, lithium concentrations in the E18 fetal plasma during 100-250 min exposure were consistently lower than in the dam’s plasma, with fetal/maternal plasma ratios ranging between 10 to 50% (Fig. 8). After long-term maternal administration of lithium by daily injections between E15-E18, which is approximately 20% of the rat 21-day gestational period, the fetal/maternal plasma concentration ratios were 30–70% (Fig. 8). Thus, rat placenta appears to substantially restrict lithium entry into fetal blood late in gestation, whether administered acutely or over several days.

There have been few studies of placental transfer of lithium in humans. In 10 patients with bipolar disorder treated with lithium throughout pregnancy, on delivery in late gestation cord blood/maternal concentration ratios were around 100% [50]. In 10 patients in a region where drinking water contained high levels of lithium (NW Argentina) in infants born at 39 week’s gestation the cord/maternal plasma ratios averaged about 150% [51]. In another study [52] in which a large number of naturally occurring elements were measured, the cord/maternal ratios for lithium showed wide variation with a mean of about 80% (n = 29). These findings perhaps suggest that in the case of lithium therapy, or ingestion of water with high naturally occurring levels of lithium, there may be unrestricted transfer between maternal and fetal blood in humans. This contrasts with the much lower ratios observed in the present study. Factors that might contribute to this difference could include differences in where blood was sampled (cord blood in humans and heart right ventricle in fetal rats), different lengths of exposure (4 days in the rats compared to continuing lithium treatment during pregnancy in humans) and species-specific differences in placental structure. The rat and human placentas are both classified as hemochorial [53, 54] but there are differences in morphology, in particular that the rat placenta has more morphological layers between the fetal and maternal circulations. In addition, there may be general differences in placental function depending on the stage of pregnancy. This is suggested by a single case example of a fetus with lithium toxicity delivered by caesarean section at 28 week’s gestation where the cord/maternal blood ratio of lithium was 86% [55], contrasting to some studies of term babies that report even greater ratios [50, 51].

### Transfer of maternally administered lithium across breast tissue and milk

Transfer of drugs through breast milk can be affected by a number of factors such as characteristics of the drug itself: molecular size, degree of Ionisation, lipid solubility and for many drugs, but not lithium, extent of plasma protein binding [56]. Maternal factors such as maternal plasma concentrations and pharmacogenomics also influence transfer [57]. Lithium concentrations in human colostrum and breast milk have been estimated to be around 50% of the maternal blood concentration [26, 52, 56] although large variations were observed (30–70% [27, 52]). These studies also reported a wide range of lithium concentrations in infant serum. Such variations perhaps relate to the time of breast feeding compared to the time of ingestion of lithium by the mother, whether as medication or in drinking water.

A key finding in the present study was detection of lithium in the plasma of pups from lactating dams that were administered long-term lithium therapy. Compared to pups directly injected with lithium (3.2 mg/kg i.p.) and sampled at steady-state (90–120 min), plasma and CSF lithium concentrations in the breastfed pups were much lower (~ 0.5 mM vs ~ 0.1 mM in plasma and ~ 0.15 mM vs ~ 0.07 mM in CSF respectively, Figs. 4 and 5). Concentrations of lithium in breast milk were not measured in this study, nor how much milk was consumed by each pup, so it was not possible to quantitate the extent of protection provided by breast tissue but by a comparison between maternal and pups’ plasma concentrations it appears that about 30% of maternal lithium gets transferred to the suckling pup.

### Mechanism of lithium entry into the CSF

Due to its very small size (hydrodynamic radius 0.079 nm, [58]) lithium would be predicted to enter the CSF from blood either by passive diffusion, transfer through ion channels or by exchange transporters where lithium substitutes for sodium ions (see “Introduction” section).

**Fig. 10:**
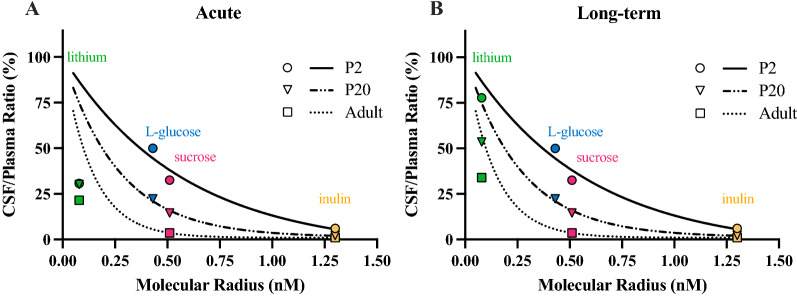
Comparison of CSF/plasma concentration ratios (%) for lithium and passive permeability markers. **A** Following acute exposure (for lithium: n = 3–10 postnatal animals, n = 1 adult; data from Fig. 4) and **B** long-term exposure in rats at different stages of development (n = 1–3 postnatal animals, n = 2 adult; data from Fig. 5. CSF/plasma ratios are plotted against the molecular radius of the markers. The curves were fitted to the data for the passive markers using single phase exponential decay non-linear regression analysis with the maximum constrained to 100% (Graphpad Prism). Note lithium values for comparison with P20 diffusion data are from P16 animals. Each point represents the average CSF/plasma ratio (%) for each age within each treatment group. Permeability data for markers other than lithium from [39].

Previous studies of blood to CSF transfer have suggested that entry of molecules is dependent on molecular size, lipid solubility and stage of development (age) with smaller molecular radius and younger age correlating with higher apparent rates of entry. This relationship has been demonstrated in a number of different species [45] including the rat [39]. However, the levels that lipid insoluble molecules reach in CSF is also heavily dependent on clearance by the turnover of CSF, which is much less in the developing brain. Thus, the much higher levels of these molecules in brain and CSF early in development, should better be referred to as an index of “apparent” permeability [36]. The smallest molecular marker previously investigated in rat was L-glucose (molecular size 180 Da, molecular radius 0.43 nm [39]) which is still much larger than the 0.079 nm hydrodynamic radius of lithium [58]. Nevertheless, if lithium predominately enters CSF by passive diffusion, it would be expected that its CSF/plasma concentration ratio would fall on the correlation lines predicted by other passively transferred markers [39]. These correlations are illustrated in Fig. 10 for postnatal pups because more data are available at these ages than for fetal stages.

In the acute exposure experiments, lithium CSF/plasma ratios fell below the levels predicted by the correlation curves for passive markers across all ages studied (Fig. 10A). In contrast, CSF/plasma ratios from the steady-state (long-term) lithium exposure experiments did fall closer to the lines predicted for passive permeability (Fig. 10B) at each stage of development investigated.

The much lower apparent entry of lithium into CSF in acute experiments, compared to its steady-state entry and compared to other markers, is not consistent with transfer by simple unrestricted passive diffusion. The CSF/plasma ratios for the passive markers used to compare with the results of present study were all measured at steady-state (i.e. approaching the equilibrium between the rates of entry into and out of CSF). Times to reach steady-state for l-glucose, sucrose and inulin were between 4 and 5 h. Based on the very small molecular size of lithium it would be expected to approach steady-state within 1.5–2 h or earlier, but the ratios reached were well below those predicted (Fig. 10A).

There are numerous ion channels and exchangers in choroid plexus epithelial cells [21]. Many of these are expressed in immature rat choroid plexus [22, 23]. However, their permeability to lithium appears not to have been investigated.

It seems unlikely that Na^+^/K^+^ ATPase activity [59] was involved as inhibition by digoxin had no effect on the entry of lithium into CSF (Fig. 9). Consistent with this is the finding that Na^+^/K^+^ ATPase expression is low in fetal and newborn rats [60].

The much lower CSF/plasma ratios for lithium in the acute experiments could perhaps be explained by restricted passive entry at the blood/CSF barrier interface. There is highly selective expression of several pore-forming claudins [1, 2, 3, 19, 22] in choroid plexus epithelium [20]. Claudin-2 has been shown to confer selective permeability to water and several cations whilst maintaining low permeability to anions and other solutes [61–63]. Lithium is able to pass through tight junctions expressing claudin-2 at permeability rates (cm/s) similar to sodium (~ 90%) and potassium (~ 75%), but different from chloride ions (> 500%). [63]. If passive transfer of cations via claudin-2 channels is a rate limiting step, slow passive entry of lithium into CSF could explain lower acute CSF/plasma ratios compared to long-term exposure. In the long-term treated animals, lithium CSF/plasma ratios were closer to the predicted steady state ratios for the age groups studied (Fig. 10B) presumably reflecting the longer time course that allows lithium to accumulate in CSF.

For comparison, steady-state CSF/plasma ratios in developing rats for sodium were reported to be just below 100% in the age range of our study [64].

**Fig. 11:**
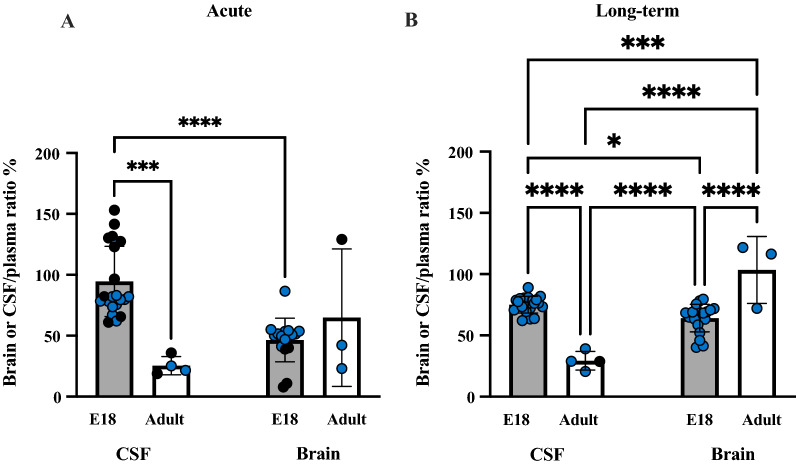
Comparison of lithium entry into the brain and CSF at different ages and treatment regimes. Brain and CSF/plasma ratios (%) of E18 fetuses and adult rats following **A** acute or **B** long-term exposure. Black points indicate samples measured with LA-ICP-MS and blue points indicate samples measured using ICP-MS. Note each point is an individual animal. In long-term treated animals, all values are significantly different to each other. Means ± SD, *p < 0.05, ***p < 0.001, ****p < 0.0001.

In the long-term treated animals, there was an age-dependent decrease in entry of lithium into CSF as illustrated in Fig. 11 where comparison is made between E18 and adult. CSF/plasma ratios decreased from 90% in the E18s to 34% in the adults. These age-related decreases are consistent with developmental increases in the rate of CSF turnover (CSF sink effect) with increasing age [65].

In a single additional long-term experiment where a much higher dose was administered to a pregnant dam at E18 (up to 10× the usual dose), lithium concentrations in the plasma increased dramatically [20–30 times], however in CSF the concentration of lithium increased only by a factor of 2 compared to concentrations found following administration of the standard dose of 3.2 mg/kg (Table 3). These findings are consistent with the long-held observation that concentrations of ions in CSF remain remarkably stable despite marked disturbances in plasma ion concentrations [66].

**Table 3:** Lithium concentrations in fetal CSF and plasma

	n	mM		CSF/plasma ratio
		Plasma	CSF	
High dose	11	1.85 ± 0.70	0.12 ± 0.04	6.9%
Standard dose	15	0.08 ± 0.04	0.06 ± 0.04	75.0%

Average concentrations of lithium in fetal CSF, plasma and CSF/plasma ratios (%) from long-term treated E18 dams. Pregnant dam from the high dose group was given up to 10 × the standard dose; the standard dose dams were given clinically relevant 3.2 mg/kg lithium, n is number of individual fetuses. Values are expressed as means ± SD.

### Entry and distribution of lithium in developing brain

The concentrations of lithium in the brains of treated dams were 0.21 and 0.24 mM (acute and long-term respectively, Table 2). These are consistent with previous studies [67, 68], which indicate there is no significant accumulation of lithium in the brain with long-term exposure. Pups in the acute treatment group had more variable concentrations in the brain, but were on average similar to the dams, mirroring concentrations in plasma. For pups receiving lithium via breast milk, concentrations of lithium in brain homogenates were much lower than in the dam, but these animals also had lower plasma concentrations (Table 2, Fig. 4). This is consistent with the suggestion that the concentration of lithium in the brain is a function of the concentration in plasma (Fig. 5).

This may suggest either a faster rate of lithium excretion from CSF or accumulation in the brain. Previous reports have described greater rates of lithium loss from CSF compared to brain tissue [68]. However, acute administration to E18 or P0 rats resulted in very low entry into the brain (Fig. 6A) in contrast to other postnatal brain/plasma ratios, whether acute or long-term, which all exceeded 50% (Fig. 6B). This suggests that before and immediately after birth, acute entry of lithium into the brain is restricted to a greater extent than after birth but this restriction can be overcome by longer-term exposure. Higher rate of transfer of lithium into the CSF but not brain following acute injection at E18 (Fig. 11) may suggest that there is limited exchange of the ion across CSF-brain barriers at this developmental stage, which is consistent with the presence of a diffusion restraint at this interface [69]. It is possible that later in development alterations to CSF-brain barrier properties and enhanced CSF flow [36, 69] may provide an environment for greater exchange of ions between CSF and brain, whereas in the early parts of development ion concentrations in the brain are more tightly controlled.

The distribution of lithium within the brain has been investigated following lithium exposure using various imaging techniques. ^6^Li(n.⍺)^3^H nuclear reaction in the presence of a dielectric particle track detector to image lithium in adult rat brain has been used [34], but the resolution was poor. Lithium‐7 nuclear magnetic resonance (^7^Li‐NMR [35]) and 3D ^7^Li magnetic resonance [70] are non-invasive in vivo methods that allowed low resolution brain mapping of lithium and regional quantitation of lithium levels in patients.

In the present study, LA-ICP-MS was used as it provides a higher resolution of anatomical features and patterns of distribution compared to MRI. Brain sections from this study revealed even distribution of lithium within the brain, but with notable accumulation in the olfactory lobes in all treated animals (Fig. 7 and Additional file 3: Fig. S2). Preferential accumulation of other metal ions in the olfactory lobe has been reported in human and rats for manganese, aluminium, nickel, zinc and cobalt [71–77]. High resolution ion imaging in the rat have provided images showing accumulation of lithium in the frontal lobe of the brain [78]. Accumulation of lithium in the olfactory lobe of rats detected in the present study could be of clinical relevance as loss of smell (hyposmia) and altered taste sensation (dysgeusia) in patients on lithium treatment [79, 80] have been reported.

Additional observations that are outside the scope of this study of lithium entry into the developing brain were that there was a prominent increase in the number of neutrophils in the blood of animals exposed to lithium (see Additional file 4: Table S6) which is consistent with leucocytosis following lithium therapy described in adults and children [81–83].

## Limitations of study

### Potential effects of kidney excretion

In the present study kidneys were left intact and this could be the explanation of the variability of lithium in the blood as the rate of excretion through the kidneys is age dependent [39]. However, nephrectomy is a very invasive technique that requires anaesthesia throughout the experimental period. This in turn would have made these experiments performed under less physiological conditions.

### Exposure time to lithium

In clinical situations patients remain on lithium treatment over prolonged periods of time. This is difficult to reproduce in animal models as rats are reluctant to consume lithium in food or water. The alternative would be to feed them forcefully but this in turn is stressful to the animals and could also result in developmental problems in the offspring. In the present study we used a “long-term” treatment 4 days of lithium i.p. injections during pregnancy and up to 18 daily i.p. injections during lactation. These periods correspond to nearly a quarter of pregnancy (in rats, gestation is 21–22 days) and most of suckling period (rats are weaned at P20). Nevertheless, it is possible that longer exposure would have resulted in more pronounced changes in lithium accumulation in the developing brain.

### Lithium in the milk

Concentrations of lithium in breast milk of lactating dams was not measured, therefore it was not possible to determine accurately how much protection is provided by the breast tissue itself. Instead, an estimate was made from the ratios between concentrations in pups versus maternal plasma levels.

## Conclusions

Lithium entry into brain and CSF was demonstrated in fetuses (E18) and postnatal animals (P0-P16/17) when lithium was administered via the mother or directly to the postnatal pups. The concentration of lithium in CSF at all ages, and in all treatment groups, was relatively similar in spite of variations in plasma levels. This indicates that the mechanisms controlling lithium entry into developing CSF are well developed early on as is known to be the case for endogenous cations such as sodium, potassium and calcium. Inhibition of Na^+^/K^+^ ATPase with a large dose of digoxin had no effect on reducing lithium entry into CSF. This indicates that other channels and ion exchange mechanisms are involved in lithium entry into the developing brain. In the pregnant animals the fetal plasma lithium concentration was only under 40% of that in maternal plasma indicating a substantial degree of placental protection at this stage of gestation. Similar restriction on lithium entry into postnatal pups via the breast milk of the lactating dam was observed. Nevertheless, in both cases lithium did enter the brain of the pups. This information on entry of lithium and its distribution in the developing brain provides background for studies of possible deleterious effects of lithium on brain development and behaviour in offspring of mothers on lithium therapy.

## Supplementary Information

**Additional file 1: Tables S1–S5.** Data on animal weights and brain, plasma, CSF lithium concentrations.

**Additional file 2: Figure S1.** Lithium dose and timing.

**Additional file 3: Figure S2.** Lithium distribution in brain.

**Additional file 4: Table S6.** Neutrophil counts in plasma.

## Data Availability

All data generated or analysed during this study are included in this published article and its Additional information files.

## Abbreviations

CSF: Cerebrospinal fluid
E: Embryonic
i.p.: Intraperitoneal.
ICP-MS: Inductively coupled plasma-mass spectrometry.
LA-ICP-MS: Laser Ablation Inductively Coupled Plasma-Mass Spectrometry.
LiCl: Lithium chloride
^7^Li‐NMR: Lithium‐7 nuclear magnetic resonance.
LoQ: Limit of quantitation.
${\text{Na}}^{+}$/${\text{K}}^{+}$ ATPase: Sodium/ potassium adenosine tri-phosphatase.
P: Postnatal
SD: Standard deviation
