# Supplementary material for: Lithium administered to pregnant, lactating and neonatal rats: entry into developing brain
Source: Fluids Barriers CNS. 2021 Dec 7;18:57. doi: 10.1186/s12987-021-00285-w (PMC8650431; doi:10.1186/s12987-021-00285-w)
Supplement: Supplementary file 2 — Additional file 2: Figure S1. Lithium dose and timing. [file 12987_2021_285_MOESM2_ESM.docx]

Figure S1

Establishment of dose and timing

| A)  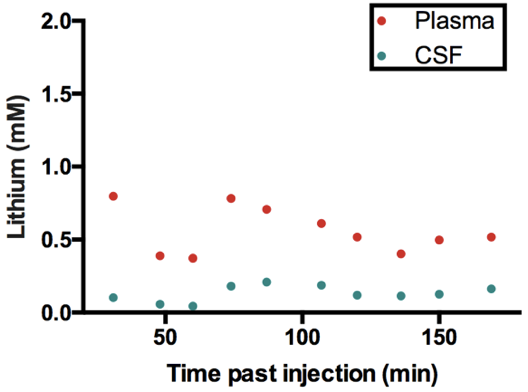 | B) 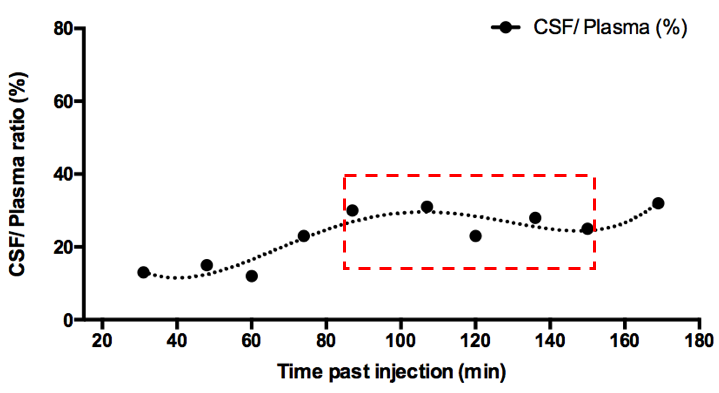 |
| --- | --- |

Concentration of lithium in plasma and CSF of P4 pups.

Each dot represents an individual pup collected 30-170 min (A). CSF/ Plasma ratio (%) for each animal is illustrated in (B). The time period when CSF/ Plasma ratios were relatively stable (90-150 min with ratios maintained at 25 $\pm$4%) boxed in (B).

NOTE: Each dot in (A) represent sample from individual animals, paired plasma and CSF from the same pup are aligned vertically on graph. CSF/ Plasma ratios (%, B) are calculated with the CSF and plasma concentration in the same animal.
