# Supplementary material for: Lithium administered to pregnant, lactating and neonatal rats: entry into developing brain
Source: Fluids Barriers CNS. 2021 Dec 7;18:57. doi: 10.1186/s12987-021-00285-w (PMC8650431; doi:10.1186/s12987-021-00285-w)
Supplement: Supplementary file 3 — Additional file 3: Figure S2. Lithium distribution in brain. [file 12987_2021_285_MOESM3_ESM.docx]

|  | E18 | P0 | P2 | P4 | P7 | P12 | P16 | Dam |
| --- | --- | --- | --- | --- | --- | --- | --- | --- |
| Acute exposure   |  | 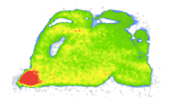 |  |  |  |  |  |  |
| 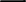 5 mm |  |  |  |  |  |  |  |  |
|  |  |  |  |  |  |  |  |  |
| Chronic exposure   |  | N/A |  |  |  |  |  | 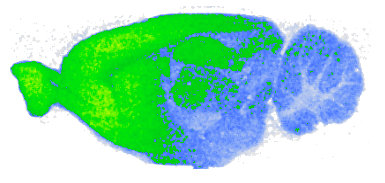 |
|  |  |  |  |  | 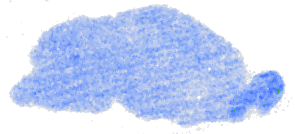 |  | 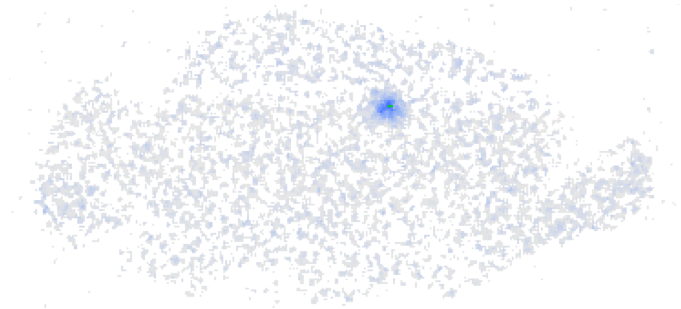 |  |
|  |  |  |  |  |  |  |  |  |

| Control pups    Lithium levels are below LoD for all control brains. |  |  |  |  |  |  |  |  |
| --- | --- | --- | --- | --- | --- | --- | --- | --- |
| Control pups  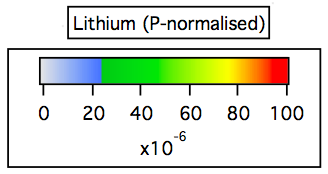  Distribution of lithium/ distribution on phosphorus (this represents presence of tissue) |  |  |  |  |  |  | 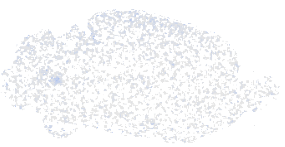 |  |
|  |  |  |  |  |  |  |  |  |

**Figure S2**. LA-ICP-MS images for lithium distribution in brain at different ages after acute or chronic lithium exposure. Sagittal brain sections were scanned using a 60 x 60 μm square ablation spot size. For control pups, lithium levels were below limit of detection (LoD) and presented grey maps represent the presence of tissue on the slide.
