# Supplementary material for: Lithium administered to pregnant, lactating and neonatal rats: entry into developing brain
Source: Fluids Barriers CNS. 2021 Dec 7;18:57. doi: 10.1186/s12987-021-00285-w (PMC8650431; doi:10.1186/s12987-021-00285-w)
Supplement: Supplementary file 4 — Additional file 4: Table S6. Neutrophil counts in plasma. [file 12987_2021_285_MOESM4_ESM.docx]

**Table S6.** Neutrophil counts in plasma of control, acute and long-term treated rats at different stages of development.


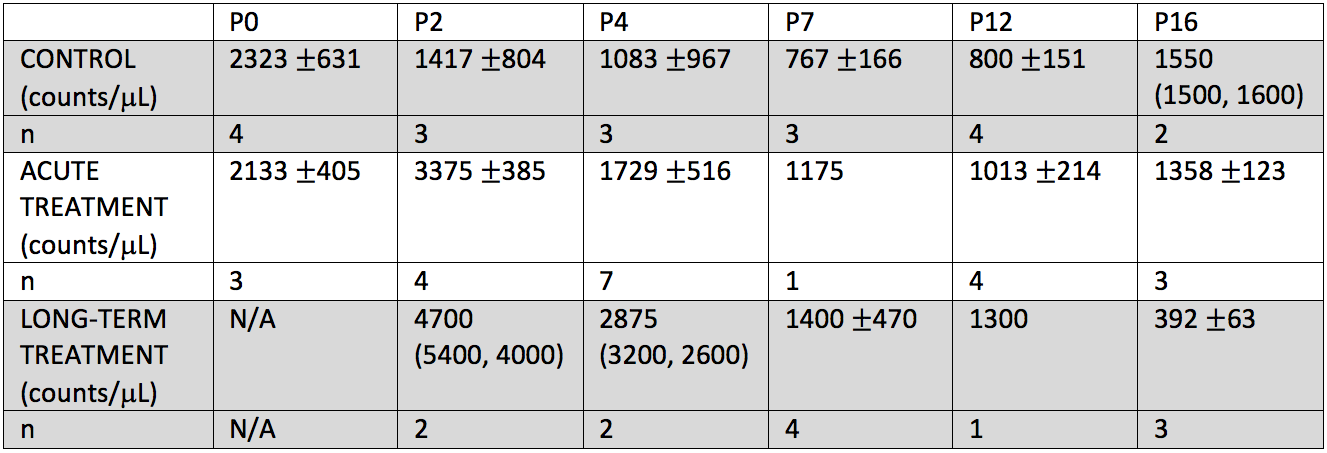


NOTE: P0 is not included in in long-term treated pups as the dam’s treatment started from the day of birth. $\pm$ represents standard deviation within each age group.
